# *QuickStats:* Death Rates* from Stroke^†^ Among Persons Aged ≥65 Years, by Sex and Age Group — National Vital Statistics System, United States, 2018

**DOI:** 10.15585/mmwr.mm6933a5

**Published:** 2020-08-21

**Figure:**
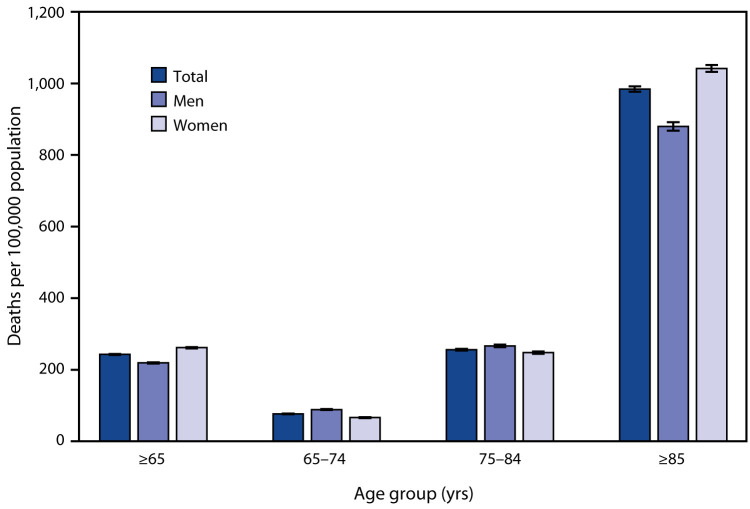
In 2018, the death rate from stroke was 242.7 per 100,000 persons aged ≥65 years. Persons aged ≥85 years had the highest death rate from stroke (984.3), followed by those aged 75–84 years (256.0) and those aged 65–74 years (76.8). For both men and women, the death rates increased with age. The death rate for women (261.6) was higher than that for men (219.0) for persons aged ≥65 years, but men had higher stroke death rates for the 65–74 and 75–84 age groups. Women aged ≥85 years had higher death rates than did men in this age group.

**Source:** National Vital Statistics System mortality data. https://www.cdc.gov/nchs/nvss/deaths.htm.

